# Assessment of community-level effects of intermittent preventive treatment for malaria in schoolchildren in Jinja, Uganda (START-IPT trial): a cluster-randomised trial

**DOI:** 10.1016/S2214-109X(18)30126-8

**Published:** 2018-04-13

**Authors:** Sarah G Staedke, Catherine Maiteki-Sebuguzi, Andrea M Rehman, Simon P Kigozi, Samuel Gonahasa, Jaffer Okiring, Steve W Lindsay, Moses R Kamya, Clare I R Chandler, Grant Dorsey, Chris Drakeley

**Affiliations:** a Department of Clinical Research, London School of Hygiene and Tropical Medicine, London, UK; b Department of Infectious Disease Epidemiology, London School of Hygiene and Tropical Medicine, London, UK; c Department of Global Health and Development, London School of Hygiene and Tropical Medicine, London, UK; d Department of Infection and Immunity, London School of Hygiene and Tropical Medicine, London, UK; e Infectious Diseases Research Collaboration, Kampala, Uganda; f Department of Biosciences, Durham University, Durham, UK; g School of Medicine, Makerere University College of Health Sciences, Kampala, Uganda; h Department of Medicine, University of California, San Francisco, CA, USA

## Abstract

**Background:**

Intermittent preventive treatment (IPT) is a well established malaria control intervention. Evidence that delivering IPT to schoolchildren could provide community-level benefits is limited. We did a cluster-randomised controlled trial to assess the effect of IPT of primary schoolchildren with dihydroartemisinin-piperaquine (DP) on indicators of malaria transmission in the community, in Jinja, Uganda.

**Methods:**

We included 84 clusters, each comprising one primary school and the 100 closest available households. The clusters were randomly assigned 1:1 to receive IPT with DP or standard care (control) by restricted randomisation to ensure balance by geography and school type. Children in intervention schools received IPT monthly for up to six rounds (June to December, 2014). We did cross-sectional community surveys in randomly selected households at baseline and in January to April, 2015, during which we measured participants' temperatures and obtained finger-prick blood smears for measurement of parasite prevalence by microscopy. We also did entomological surveys 1 night per month in households from 20 randomly selected IPT and 20 control clusters. The primary trial outcome was parasite prevalence in the final community survey. The primary entomological survey outcome was the annual entomological inoculation rate (aEIR) from July, 2014, to April, 2015. This trial is registered at ClinicalTrials.gov, number NCT02009215.

**Findings:**

Among 23 280 students registered in the 42 intervention schools, 10 079 (43%) aged 5–20 years were enrolled and received at least one dose of DP. 9286 (92%) of 10 079 received at least one full course of DP (three doses). Community-level parasite prevalence was lower in the intervention clusters than in the control clusters (19% *vs* 23%, adjusted risk ratio 0·85, 95% CI 0·73–1·00, p=0·05). The aEIR was lower in the intervention group than in the control group, but not significantly so (10·1 *vs* 15·2 infective bites per person, adjusted incidence rate ratio 0·80, 95% CI 0·36–1·80, p=0·59).

**Interpretation:**

IPT of schoolchildren with DP might have a positive effect on community-level malaria indicators and be operationally feasible. Studies with greater IPT coverage are needed.

**Funding:**

UK Medical Research Council, UK Department for International Development, and Wellcome Trust.

## Introduction

Over the past decade, reductions in malaria burden have been documented worldwide after heavy investment in control measures.^1^ However, in sub-Saharan Africa, gains in malaria control in countries with the highest transmission have not been consistent.^2^ In Uganda, the burden of malaria remains high, despite upscaled distribution of long-lasting insecticide-treated nets and use of artemisinin-based combination therapies to treat symptomatic malaria.^3^ In areas of high malaria transmission in Uganda, indoor residual spraying is substantially more effective than long-lasting insecticide-treated nets,^4^ but is expensive and difficult to sustain.^5^ Innovative malaria control efforts are needed in areas with high, perennial transmission.

Intermittent preventive treatment (IPT) is a well established malaria control intervention, recommended for use in pregnant women and infants in specific settings, and for children younger than 5 years in areas of seasonal transmission. The malaria burden in school-aged children has been underappreciated,^6^ but a wealth of evidence from Uganda and elsewhere suggests that IPT of malaria in schoolchildren provides substantial health benefits and might improve cognitive function.^7^, ^8^, ^9^, ^10^ Additionally, parasite prevalence is typically highest in school-aged children (although age ranges vary with transmission intensity), who serve as reservoirs of infection for the onward transmission of malaria to mosquitoes.^11^ Targeted use of chemoprevention in school-aged children will benefit individual children^7^ and might decrease malaria transmission by reducing the infectious reservoir, which would benefit the community.^9^ However, little evidence is available of community-level benefits.

**Research in context**

**Evidence before this study**

We searched PubMed for original articles published in English between Jan 1, 2000, and Oct 1, 2017, with the term “intermittent, preventive treatment AND malaria OR Plasmodium falciparum AND schoolchildren NOT infant NOT pregnancy”. We assessed titles and abstracts and found no studies that had investigated the effects of intermittent preventive treatment (IPT) for malaria in schoolchildren on malaria transmission at the community level. One systematic review had assessed the efficacy and safety of IPT in schoolchildren. This review included five studies, including four individually randomised trials and one cluster-randomised trial, which were done in 2002–12 in Kenya, Mali, and Uganda, but none reported population-level health effects. Three later studies of school-based malaria interventions included one individually randomised trial of IPT for malaria and helminths in Ghana, one individually randomised trial of IPT for malaria in the Democratic Republic of Congo, and one cluster-randomised trial of IPT for malaria plus distribution of long-lasting insecticide-treated nets in Mali. Again, none assessed population-level outcomes. One stepped-wedge, cluster-randomised trial in Senegal assessed community-level outcomes but with seasonal malaria chemoprevention in schoolchildren and not IPT. In that trial, children younger than 10 years received amodiaquine and sulfadoxine-pyrimethamine, administered by community health workers via health posts. No reduction in all-cause mortality (the primary outcome) was seen, but the incidence of confirmed malaria in community residents too old to receive seasonal malaria chemoprevention reduced significantly by 26%. These results were encouraging, but the intervention, method of assessment, and outcome measures differed substantially from those in our trial, precluding direct comparisons.

**Added value of this study**

This study in Jinja, Uganda, makes an important contribution to the limited evidence on use of IPT as a tool to reduce malaria transmission. The use of dihydroartemisinin-piperaquine for IPT of malaria in schoolchildren was associated with reductions in parasite prevalence and annual entomological inoculation rate, but these were of borderline significance. Intervention coverage, however, was lower than expected.

**Implications of all the available evidence**

Evidence on the effects of providing IPT of malaria to schoolchildren is limited, but promising. Schoolchildren are major contributors to the infectious reservoir, and providing IPT in schools offers an operationally attractive and potentially sustainable intervention that could be integrated with currently deployed malaria control methods. Additional studies are needed to explore these findings further, including assessments of the population-level effects with greater intervention coverage.

In the School-based Treatment with ACTs to Reduce Transmission of malaria (START-IPT) trial, we investigated whether IPT for malaria with dihydroartemisinin-piperaquine (DP) in schoolchildren would affect community-level indicators of malaria transmission in Jinja district, Uganda. DP is highly efficacious and well tolerated, and is more effective than other artemisinin-based combination therapies in preventing new infections because of the long half-life of piperaquine, making it an attractive option for IPT.^12^ We hypothesised that malaria transmission, as measured by the prevalence of asexual parasitaemia in the community and the annual entomological inoculation rate (aEIR), would be reduced by the intervention compared with no IPT.

## Methods

### Study design

START-IPT was a cluster-randomised controlled trial. We obtained ethics approval from the Ugandan National Council for Science and Technology (reference HS 1530); Makerere University School of Medicine Research and Ethics Committee, Kampala, Uganda (SBS 145); the London School of Hygiene & Tropical Medicine Ethics Committee, London, UK (6509); the School of Biological and Biomedical Sciences Ethics Committee, Durham University, Durham, UK; and the University of California, San Francisco Committee on Human Research, San Francisco, CA, USA (074826). Sponsorship and insurance was provided by the London School of Hygiene & Tropical Medicine Clinical Trials Sub-Committee (reference QA380). The trial was overseen by an independent data and safety monitoring board and a trial steering committee. We obtained verbal consent from schools and written consent from students and community residents (with assent from children aged ≥8 years).

### Study site

Jinja district is an area of perennial malaria transmission in eastern Uganda. The district has 11 subcounties that range from periurban areas of Jinja town (including Walukuba, the only area with an estimated aEIR [3·8 infective bites per person in 2011–12^13^], but which is not representative of the whole district) to rural areas in the north, where malaria transmission is more intense. All subcounties were eligible for inclusion in the trial.

### Clusters and randomisation

**Figure 1:**
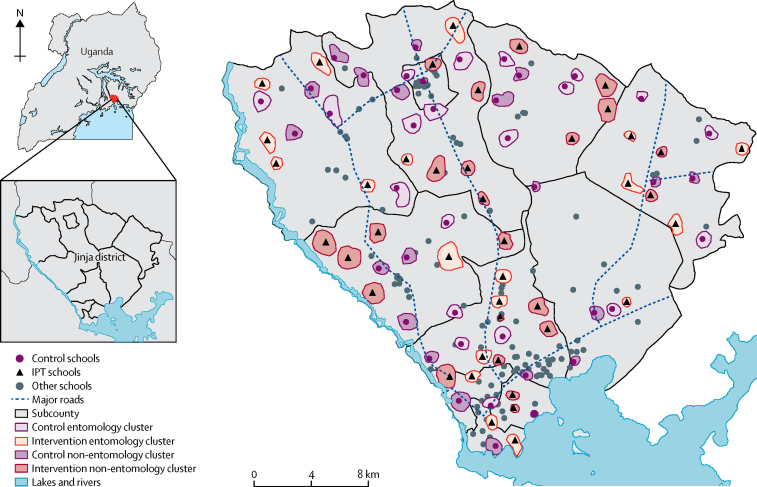
Map of study area The study included 84 clusters, each including one primary school and the closest available 100 surrounding households. IPT=intermittent preventive treatment.

We defined cluster boundaries with digitally enumerated maps (figure 1, appendix pp 1–2). Each cluster included one primary day school and the 100 closest available surrounding households. To minimise contamination, we implemented a buffer zone of 500 m between clusters. If catchment areas for more than one school overlapped, one school was selected: if a cluster contained public and private schools, public schools were prioritised, and if schools were the same type, one was chosen randomly. Clusters were assigned 1:1 to receive IPT or standard care (services typically provided by the Ministries of Health and Education; control) in a parallel design. We used restricted randomisation to ensure balance across clusters for geographical location by subcounty and school type (public or private). A simulated data series of 15 000 allocations was generated by the trial statistician with Stata/SE (version 12.1), which produced a list of 5735 potential allocations that met the restriction criteria. Study personnel enrolled primary schools after cluster randomisation without masking of study group allocation.

### IPT intervention

Study personnel met with stakeholders in health and education at the national and district levels to build awareness and support for the trial. An information sheet described the study, and verbal consent to participate was obtained from headteachers of schools after randomisation. Copies of the school registers were obtained from schools in the intervention clusters.

Study personnel initially reviewed eligibility of schoolchildren in the intervention cluster schools with parents and guardians. Eligibility criteria were ability to locate a parent or guardian, being enrolled in an intervention school, age 5 years or older, no known allergy to DP, no menarche, no history of cardiac problems or fainting, no family history of long QT syndrome, not currently taking medications known to prolong the QT interval, and willingness of the parent or guardian to provide written informed consent. If these criteria were met, study personnel interviewed children individually at school to assess the final eligibility criteria: ability to locate the student, no menarche, weight 11 kg or greater, and provision of written assent by student if aged 8 years or older. After screening, students underwent a brief physical examination, including measurement of temperature (tympanic membrane), height, and weight. We recorded participants' fingerprints to facilitate future identification for DP treatment.

The DP preparation was full-strength Duo-Cotexcin tablets (Beijing Holley-Cotec Pharmaceuticals, Beijing, China), containing 40 mg dihydroartemisinin and 320 mg piperaquine and taken orally. IPT was delivered to students in intervention schools by study personnel, with doses given once daily for 3 days per month (one round of treatment), according to weight-based guidelines (appendix p 3), for up to six rounds of treatment. All treatments were directly observed and recorded.

To assess safety we monitored all intervention participants for serious adverse events, and a subset of participants selected by convenience sampling for cardiac monitoring. The relationship of serious adverse events to DP was assessed by study staff, and all serious adverse events were reported to the institutional review boards and the trial's data and safety monitoring board. Detailed results on safety will be reported separately.

### Assessments

Cross-sectional community surveys were done in households randomly selected from each cluster at baseline (March to June, 2014) and after the intervention (January to April, 2015). The sampling frame for the baseline survey was generated from the digital enumeration done to generate clusters, and for the final survey from a census of clusters. Each survey was done in a different random sample.

Study personnel visited households for both community surveys to identify those with an adult resident available to do the survey who met the following eligibility criteria: a usual resident present on the night before the survey, age 18 years or older, and agreement to provide written informed consent. Households were excluded if the dwelling could not be found or was vacant or if no adult resident was at home on more than three visits. If selection criteria were met, a household questionnaire was administered to the head of the household or their designate to gather information on all residents, ownership and use of all bednets, and proxy indicators of wealth.

The target sample sizes of age groups varied because of age-related differences in parasite prevalence. Thus, recruitment of household residents into the community clinical surveys was stratified by age. For the baseline survey, all members of a household were eligible for inclusion. For the final survey, all members of a household aged 4 months to younger than 5 years or older than 15 years were eligible for inclusion, but for residents aged 5–15 years, only one per household could be included, and was chosen at random by random number tables. To participate in the clinical survey, household residents were reviewed for selection criteria, including being a usual resident of the household on the night before the survey, ability to locate the resident, appropriate age, provision of written informed consent for adults or parents or guardians of children, and provision of written assent from children aged 8 years and older.

At the time of the survey, we measured each participant's temperature and obtained a finger-prick blood sample that was used to prepare a thick blood smear to measure haemoglobin concentration with a portable spectrophotometer (HemoCue, Ängelholm, Sweden) in children younger than 5 years. Samples were stored on filter paper for future molecular testing. Rapid diagnostic testing was done with CareStart Malaria HRP2 for *Plasmodium falciparum* (ACCESSBIO, Somerset, NJ, USA) in participants who had fever (tympanic membrane temperature ≥38·0°C) or history of fever in the previous 48 h.

We did an entomological survey in 200 households, five per cluster in 20 of the IPT clusters and five per cluster in 20 of the control clusters. The clusters and households were randomly selected before the survey started. Selection criteria were as follows: the ability to locate the household; residence in the subcounty for the previous 6 months; and provision of written informed consent by the head of the household (or designate). Participating households were sampled one night per month with miniature Centers for Disease Control and Prevention light traps (Model 512, John W Hock Company, Gainesville, FL, USA). Traps were positioned 1 m above the floor at the foot end of the bed where a person slept under an insecticide-treated net.

### Laboratory procedures

Thick blood smears were stained with 2% Giemsa for 30 min and read by experienced laboratory technologists who were unaware of study group assignments. Parasite and gametocyte densities were calculated from thick blood smears by counting the number of asexual parasites and gametocytes, respectively, per 200 leucocytes (or per 500, if the count was less than ten parasites or gametocytes per 200 leucocytes), assuming a leucocyte count of 8000 cells per μL (8·0 × 10^9^/L). We classified the result as negative if examination of 100 high-power fields revealed no asexual parasites or gametocytes. For quality control, all slides were read by a second microscopist, and a third reviewer designated discrepant readings.

DNA was extracted from dried blood samples on filter papers by standard methods with Chelex 100 Resin (Bio-Rad, Hercules, CA, USA) and analysed by loop-mediated isothermal amplification (LAMP) for detection of *P falciparum* parasites. Mosquitoes were identified taxonomically to species level where possible. Identification of anophelines was based on established morphological criteria, and members of the *Anopheles gambiae* complex were identified by PCR.^13^ Sporozoites were identified with ELISA.^13^

### Outcomes

The primary outcome was prevalence of asexual parasitaemia in the final community survey, as measured by microscopy of individual blood smears. Secondary outcomes were prevalence of parasitaemia by microscopy and LAMP, prevalence of gametocytaemia, prevalence of anaemia,^14^ and mean haemoglobin concentration in the final community survey. The primary outcome for the entomology survey was aEIR, estimated from July, 2014, when the intervention began, to April, 2015. Secondary entomological outcomes were sporozoite rate and vector density per house.

### Statistical analysis

The target sample size for the baseline survey was based on age-stratified estimates of parasite prevalence from Walukuba subcounty in Jinja district.^15^ The prevalence of parasitaemia at baseline was assumed to vary with age and was estimated to be 4% in children younger than 5 years, 17% in children aged 5–15 years, and 9% in individuals older than 15 years. To detect a relative reduction of 35% in the intervention group compared with the control group in each age group and in 22% of respondents overall, we calculated that we would need to recruit 119 individuals per cluster (minimum total 9996), including 73 children younger than 5 years, 15 children aged 5–15 years, and 31 individuals older than 15 years (appendix pp 4–5).

Sample size calculations for the final community survey were informed by data collected in the baseline survey. Calculations accounted for correlation among clusters by calculating the coefficient of variation (k).^16^ We conservatively assumed a k value of 0·5. The number of clusters was fixed at 84. Assuming significance of 5% and power of 80%, we calculated that 105 individuals per cluster (minimum total 8820) would allow detection of a relative reduction in parasite prevalence of 29%, based on 21% prevalence in the control group. This estimate was supported by mathematical modelling estimates^17^ that we adapted to simulate the START-IPT trial design and setting. This relative difference in parasitaemia would correspond to an absolute difference in parasite prevalence of 6% (21% *vs* 15%). Additionally, we weighted the sample sizes of age groups to ensure 80% power to detect a relative difference in parasite prevalence of 30·0% between study groups for children younger than 5 years (n=64), 34·5% for children aged 5–15 years (n=14), and 40% for individuals older than 15 years (n=27).

For the entomological survey, we calculated a k value of 0·57 for variation between houses, based on preliminary data from Uganda (Dorsey G, unpublished). With this level of variation, we calculated that five households in 20 clusters in each study group would be sufficient to detect a 50% reduction in aEIR in the IPT group with 80% power and 5% significance.^16^

We did all analyses by intention to treat. Thus, all community residents were classified as participating in the intervention or control irrespective of whether they (or their children) received the intervention. For all analyses, we used statistical methods that allowed for within-cluster correlations, and analysed data with STATA/IC version 12.1. All outcomes were assessed at the individual level because of the large number of clusters per study group.

For binary prevalence outcomes, we used generalised linear Poisson models with log link function. We compared the effects of the intervention between groups with prevalence risk ratios (RRs) and 95% CIs. The probability of selection for the final community survey was related to the sampling frame, which was determined by the expected parasite prevalence in the age categories younger than 5 years, 5–15 years, and older than 15 years. Therefore, the age structure of the study population was not representative of the community population overall. To account of this discrepancy, we assigned individuals one of three weights based on their age, calculated as the inverse of the percentage of people in each age group in the census survey. Population estimates of prevalence were obtained with svy commands in STATA, with the cluster as the primary sampling unit.

The annual human biting rate was calculated as total number of female *Anopheles* spp mosquitoes captured/number of house-nights of collection × 365·25 days per year. The sporozoite rate was calculated as the number of mosquitoes positive for sporozoites / the number of mosquitoes tested. The aEIR was the product of the annual human biting rate and the sporozoite rate. We compared aEIRs between study groups with negative binomial regression (to account for overdispersion), with random effects for clusters and repeated measures. The number of mosquitoes positive for sporozoites was used as the outcome with an offset variable of the natural log of the number of house-nights of capture and having a coefficient constrained to 1. Households selected for the entomological survey were randomly selected and, therefore, needed no weighting. We did a subgroup analysis to assess whether the effect of the intervention changed over time in three time periods after the intervention was first implemented: 1–3 months (July to September, 2014), 4–6 months (October to December, 2014), and 7–10 months (January to April, 2015). This trial is registered at ClinicalTrials.gov, number NCT02009215.

### Role of the funding source

The funder of the study had no role in study design, data collection, data analysis, data interpretation, or writing of the report. The corresponding author had full access to all the data in the study and had final responsibility for the decision to submit for publication.

## Results

**Figure 2:**
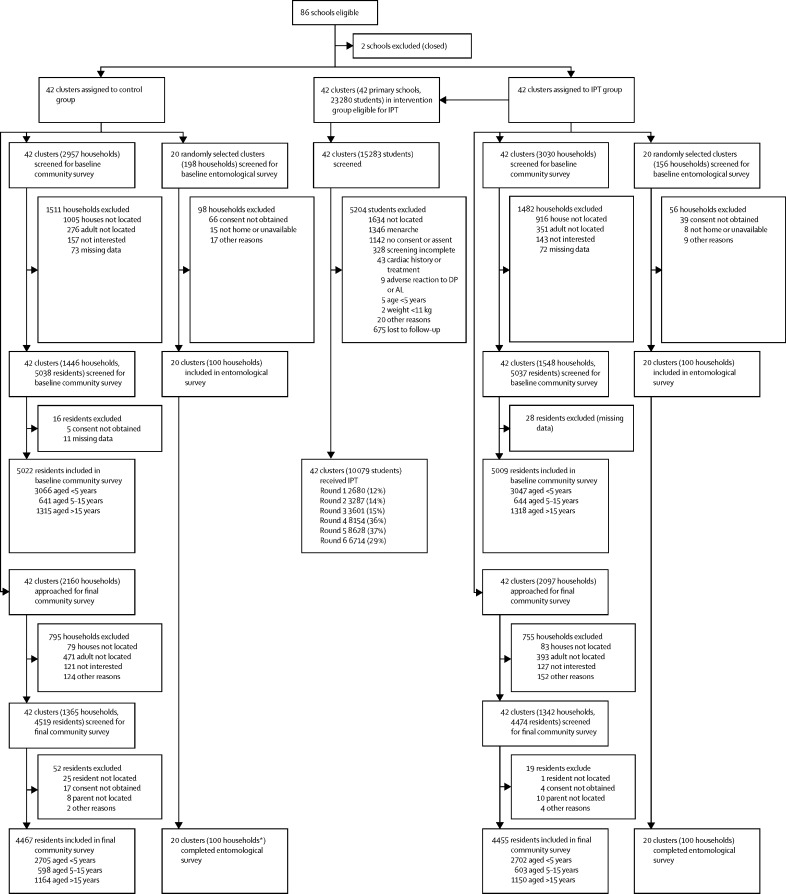
Trial profile IPT=intermittent preventive treatment. DP=dihydroartemisinin-piperaquine. AL=artemether-lumefantrine. *One household was excluded and replaced with a newly recruited household.

Of 86 eligible clusters 84 were included in the study (figure 2): 42 were assigned to the intervention group and 42 to control. 72 schools were public and 12 were private schools. In the census of cluster households for the final community survey, 44 440 individuals were enumerated within the study area, among whom 7675 (17%) were children younger than 5 years, 14 704 (33%) were children aged 5–15 years, and 22 061 (50%) were individuals older than 15 years of age. 11 336 (26%) of 44 440 were reported to attend primary school, among whom 5480 (48%) attended a school within a cluster boundary (12% of the cluster population). Population sizes did not differ between clusters surrounding public and private schools.

The baseline community survey was done from March to June, 2014, and included 2994 households (median per cluster 35, range 19–56) and 10 031 participants (119, 107–123; figure 2). Characteristics were similar across study groups (table 1). 2962 (99%) households reported owning at least one insecticide-treated net, and 7573 (76%) participants reported having slept under a bednet during the night before the survey. Prevalence of parasitaemia was highest in children aged 5–15 years, followed by children younger than 5 years, and individuals older than 15 years (table 1). Characteristics of clusters were also similar across study groups. In each study group, 36 (86%) clusters contained a public school and six (14%) private schools. Baseline cluster parasite prevalence was similar (median 25·4%, IQR 13·4–33·6 in the intervention group and 25·2%, 10·1–31·4 in the control group). The entomological survey was completed in all 200 households selected (figure 2). Characteristics of households in the entomological survey did not differ between the study groups (table 2).

**Table 1:** Characteristics of households and participants in baseline community survey

			**Control group**	**Intervention group**
**Households**				
Total households interviewed			1446	1548
Total residents			8348	8640
Mean number of residents per household			5·8 (0·14)	5·6 (0·11)
Have electricity			326 (23%)	316 (20%)
Have mobile phone			1225 (85%)	1354 (88%)
Head of household education*				
	No education		164 (11%)	194 (13%)
	Primary school (P1–6)		702 (49%)	633 (41%)
	Secondary school (S1–6)		422 (29%)	531 (34%)
	Certificate, diploma, university		143 (10%)	167 (11%)
Household wealth index†				
	1 (poorest)		296 (21%)	309 (20%)
	2		279 (19%)	312 (20%)
	3		259 (18%)	338 (22%)
	4		304 (21%)	294 (19%)
	5 (least poor)		305 (21%)	292 (19%)
Own at least one ITN			1429 (99%)	1533 (99%)
Nets per household			2·3 (0·12)	2·4 (0·12)
**Survey participants**				
Total enrolled				
	<5 years		3066	3047
	5–15 years		641	644
	>15 years		1315	1318
Age (years)				
	<5 years		2·5 (0·03)	2·5 (0·03)
	5–15 years		9·5 (0·16)	9·1 (0·14)
	>15 years		33·6 (0·62)	33·4 (0·64)
Male/female participants				
	<5 years		1564 (51%)/1502 (49%)	1563 (51%)/1484 (49%)
	5–15 years		346 (54%)/295 (46%)	346 (54%)/298 (46%)
	>15 years		863 (66%)/452 (44%)	887 (67%)/431 (33%)
Slept under an ITN the previous night				
	<5 years		2383 (78%)	2250 (74%)
	5–15 years		449 (70%)	449 (70%)
	>15 years		1028 (78%)	1014 (77%)
Febrile (≥38·0°C or history of fever in previous 48 h)				
	<5 years		2041 (67%)	2049 (67%)
	5–15 years		339 (53%)	345 (54%)
	>15 years		600 (46%)	525 (40%)
Rapid diagnostic test				
	Done			
		<5 years	2009 (98%)	2023 (99%)
		5–15 years	334 (99%)	330 (96%)
		>15 years	572 (95%)	488 (93%)
	Positive			
		<5 years	973 (48%)	978 (48%)
		5–15 years	189 (57%)	170 (52%)
		>15 years	173 (30%)	129 (26%)
Haemoglobin concentration (g/L)				
	<5 years		114 (0·8)	113 (0·9)
	5–15 years		123 (0·8)	121 (0·9)
	>15 years		130 (0·8)	130 (0·9)
Anaemia‡				
	<5 years		1057 (35%)	1150 (38%)
	5–15 years		157 (24%)	187 (29%)
	>15 years		351 (27%)	327 (25%)
Parasitaemia (blood slide positive)				
	<5 years		809 (26%)	806 (27%)
	5–15 years		207 (32%)	208 (32%)
	>15 years		167 (13%)	180 (14%)
Gametocytaemia (blood slide positive)				
	<5 years		230 (8%)	238 (8%)
	5–15 years		40 (6%)	38 (6%)
	>15 years		19 (1%)	14 (1%)

Data are number, number (%), or mean (SE). ITN=insecticide-treated net.

* Data missing for 15 (1%) control households and 23 (2%) intervention households.

† Data missing for three (<1%) control households and three (<1%) intervention households.

‡ Defined as haemoglobin concentration <110 g/L in children <5 years, <115 g/L in children aged 5–11 years, <120 g/L in children aged 12–14 years and girls and women ≥15 years, and <130 g/L in boys and men aged ≥15 years.

**Table 2:** Characteristics of households in the baseline entomology survey, April to June, 2014

	**Control**	**Intervention**
Total clusters	20	20
Total households	101*	100
Mean number of residents per household (SE)	5·30 (0·24)	5·25 (0·42)
Number of households with floor mainly made of floor bricks, cement, or stones (%)	48 (48%)	50 (50%)
Number of households with roof mainly made of metal (%)	98 (97%)	93 (93%)
Number of households with external walls mainly made of burnt bricks with plaster (%)	69 (68%)	71 (71%)
Human biting rate†	2330	2206
Sporozoite rate‡	2·8%	2·4%
aEIR§ (cluster median, IQR)	56·3 (18·3, 0–73·1)	61·5 (0, 0–36·5)

aEIR=annual entomological inoculation rate.

* One household withdrew consent in 2014, and was replaced.

† Total number of mosquitoes captured / number of nights of collection × 365·25.

‡ Number of mosquitoes positive for sporozoites  / number of mosquitoes tested.

§ Product of the human biting rate and sporozoite rate.

We recruited children to receive IPT from March to December, 2014 (figure 2). 23 280 students were listed on the registers for the 42 intervention schools (mean per school 554, range 131–1521). 7997 (34%) were not screened because a parent or guardian could not be located and an additional 5204 (34%) were excluded during screening. 89 823 doses of DP were administered to 10 079 (43%) children from June to December, 2014, among whom 9286 (92%) received at least one full course of DP. Given the rolling recruitment, the number of children enrolled in the intervention and available for treatment per round increased during the study (table 3), but the proportion who received DP in each round varied because of school schedules and holidays. IPT treatment was greatest in round 5 (Oct 20 to Nov 14, 2014), when 8628 (37%) of 23 280 children received at least one dose of DP and 7007 (30%) received all three doses. The proportion of children enrolled in the intervention who received all three doses for a given round of IPT was high, ranging from 69% (2254 of 3287 in round two) to 84% (3032 of 3601 in round three; table 3).

**Table 3:** Enrolment into the intermittent preventive treatment intervention group in 2014, by round and treatment coverage

	**Enrolled (n=23 280)**		**Received any dose of DP**		**Received a full course of DP (three doses)**	
	Number (%)	Median (IQR) proportion per cluster (%)	Number (%)	Median (IQR) proportion per cluster (%)	Number (%)	Median (IQR) proportion per cluster (%)
Round 1 (June 30–July 25)	2680 (12%)	13% (9–19)	2680 (12%)	13% (9–19)	2210 (9%)	11% (8–16)
Round 2 (July 28–Aug 22)	3290 (14%)	14% (9–20)	3287 (14%)	14% (9–20)	2254 (10%)	9% (6–15)
Round 3 (Aug 25–Sept 19)	3601 (15%)	15% (11–24)	3601 (15%)	15% (11–24)	3032 (13%)	13% (7–18)
Round 4 (Sept 22–Oct 17)	8159 (35%)	36% (29–49)	8154 (35%)	36% (29–49)	6778 (29%)	32% (24–40)
Round 5 (Oct 20–Nov 14)	8634 (37%)	41% (33–49)	8628 (37%)	41% (33–49)	7007 (30%)	33% (23–39)
Round 6 (Nov 17–Dec 12)	6714 (29%)	33% (22–41)	6714 (29%)	33% (22–41)	5110 (22%)	24% (15–33)

23 280 indivuals were registered in the 42 intervention schools. DP=dihydroartemisinin-piperaquine.

17 serious adverse events were reported, including two deaths (one from a road traffic accident and one from a tetanus infection, both unrelated to DP; table 4). Among the remaining 15 serious adverse events, two—an allergic skin reaction and a case of weakness and loss of consciousness after severe abdominal pain that was attributed to hypoglycaemia—were judged to be possibly related to DP.

**Table 4:** Serious adverse events

	**Age (years)**	**Sex**	**Maximum severity**	Related to DP*	**Event outcome**
Death after motor vehicle accident	8	Girl	Life threatening	No	Death
Death after tetanus infection	7	Boy	Life threatening	No	Death
Allergic dermatitis (generalised papular rash)	10	Boy	Severe	Possibly	Resolved, without sequelae
Weakness and loss of consciousness after severe abdominal pain	12	Girl	Severe	Possibly	Resolved, without sequelae
Acute gastritis and upper respiratory tract infection	8	Girl	Severe	Unlikely	Resolved, without sequelae
Acute gastritis with moderate dehydration	8	Girl	Severe	Unlikely	Resolved, without sequelae
Severe body weakness†‡	9, 11, 12, and 13	All girls	All severe	All unlikely	All resolved, without sequelae
Malaria with upper respiratory tract infection	8	Boy	Severe	Unlikely	Resolved, without sequelae
Malaria with gastroenteritis	12	Boy	Severe	Unlikely	Resolved, without sequelae
Multiple fractures after road traffic accident	8	Girl	Severe	No	Resolved, without sequelae
Malaria	7	Boy	Severe	No	Resolved, without sequelae
Malaria, upper respiratory tract infection, and oral candidiasis	7	Boy	Severe	No	Resolved, without sequelae
Malaria and suspected bacteraemia	7	Girl	Severe	No	Resolved, without sequelae
Epigastric pain attributed to peptic ulcer disease‡	13	Girl	Moderate	Unlikely	Resolved, without sequelae

DP=dihydroartemisinin-piperaquine.

* Study staff assessed serious adverse events to determine the suspected relationship to DP.

† Four study participants from the same school had severe body weakness on the same day after mass administration of azithromycin for trachoma.

‡ Two serious adverse events were seen in one participant.

The prevalence of parasitaemia by microscopy in the final community survey was marginally lower in the intervention group than the control group (table 5). Results were similar when stratified by age. The overall coefficient of variation of prevalence between clusters was k=0·72 (mean 0·21 [SD 0·15]), and was lowest for children aged 5–15 years in control clusters (k=0·60), followed by individuals older than 15 years in control clusters (k=0·71), and highest for children younger than 5 years in control clusters (k=0·73). Parasite prevalence was higher when microscopy results were supplemented by LAMP testing (weighted by the inverse of the population proportion and the probability of LAMP sampling), particularly in children aged 5–15 years. In all age groups, parasite prevalence by microscopy and LAMP was lower in the intervention group than the control group, although these differences were not significant in the adjusted analysis except for children aged 5–15 years (table 5).

**Table 5:** Effect of intermittent preventive treatment on parasitaemia in the final community survey

		**Number positive of total respondents**	**Prevalence**	**Crude risk ratio (95% CI)**	**p value**	Adjusted risk ratio*(95% CI)	**p value**
**Microscopy**							
All ages†							
	Control	978 of 4467	23·1%	1		1	
	Intervention	791 of 4455	19·0%	0·82 (0·61–1·12)	0·21	0·85 (0·73–1·00)	0·05
<5 years							
	Control	638 of 2705	23·6%	1		1	
	Intervention	533 of 2702	19·7%	0·84 (0·61–1·15)	0·27	0·86 (0·73–1·02)	0·09
5–15 years							
	Control	196 of 598	32·8%	1		1	
	Intervention	150 of 603	24·9%	0·76 (0·55–1·04)	0·08	0·78 (0·60–1·00)	0·05
>15 years							
	Control	144 of 1164	12·4%	1		1	
	Intervention	108 of 1150	9·4%	0·76 (0·53–1·09)	0·14	0·79 (0·59–1·05)	0·11
**Microscopy plus LAMP**							
All ages‡							
	Control	1419 of 2410	42·1%	1		1	
	Intervention	1192 of 2312	37·5%	0·89 (0·72–1·10)	0·28	0·93 (0·84–1·04)	0·18
<5 years							
	Control	760 of 1156	40·4%	1		1	
	Intervention	657 of 1077	37·1%	0·92 (0·73–1·16)	0·47	0·96 (0·84–1·09)	0·50
5–15 years							
	Control	341 of 598	54·7%	1		1	
	Intervention	281 of 603	43·8%	0·80 (0·67–0·96)	0·02	0·84 (0·73–0·97)	0·02
>15 years							
	Control	318 of 656	42·7%	1		1	
	Intervention	254 of 632	34·6%	0·81 (0·64–1·02)	0·07	0·85 (0·73–1·00)	0·06
**Prevalence of gametocytaemia**							
Control		217 of 4467	5·3%	1		1	
Intervention		207 of 4455	5·1%	0·95 (0·64–1·40)	0·78	1·00 (0·73–1·37)	0·99
**Prevalence of anaemia**§							
Control		1083 of 2705	40·0%	1		1	
Intervention		1040 of 2702	38·5%	0·96 (0·80–1·15)	0·67	1·03 (0·87–1·23)	0·72

LAMP=loop-mediated isothermal amplification.

* Adjusted for baseline community parasite prevalence (0–13%, >13–25%, >25–33%, or >33%), sex, individual bednet use, school type, subcounty, and socioeconomic status quintiles, eaves status (all closed, some closed, or all open), window screening status (all, some, or none screened), and latitude (<4°, 4 to <5°, or >5°).

† Overall prevalence was weighted by the inverse of the percentage of people counted in each age group in the cluster census (17·3% for <5 years, 33·1% for 5–15 years, and 49·6% for >15 years).

‡ Overall prevalence weighted by the inverse of the percentage of the population (17·3% of children <5 years, 33·1% of children aged 5–15 years, and 49·6% of people >15 years) multiplied by the probability of being sampled for LAMP (the product of the percentage negative by microscopy, because only negative samples were eligible for LAMP testing) and an age-related probability. Sampling was done in 25% of from children <5 years, 100% of children aged 5–15 years, and 50% of people >15 years.

§ Only measured in children <5 years and was defined as haemoglobin concentration <110 g/L.

In the entomological survey, 27 034 mosquitoes were collected over 1936 nights, of which 6651 were *Anopheles* spp. 6322 (95%) of anophelines collected were *A gambiae* sensu lato, 277 (4%) *A funestus*, 51 (1%) *A arabiensis*, and one (<0·5%) another species. The number of female *Anopheles* spp mosquitoes, the human biting rate, sporozoite rate, and aEIR were all lower in the intervention group than in the control group (table 6). Overall, the aEIR was 10·1 infectious bites per person (cluster median 0, IQR 0–14·5) in the intervention clusters versus 15·2 (7·4, 0–19·6) in the control clusters, but varied over time. During the period of peak delivery of DP (months 4–6 of the IPT intervention in October to December, 2014), the aEIR and sporozoite rate were lower in the intervention group than in the control. The overall coefficient of variation of the prevalence between clusters was k=1·69.

**Table 6:** Effect of the intermittent preventive treatment intervention on entomological inoculation rate in the entomology survey, stratified by time

		**Number of female *Anopheles* spp collected**	**Number of nights of collection**	**Annual density of *Anopheles* spp per household**	**Number of mosquitoes positive for sporozoites**	**Sporozoite rate**	aEIR*	**Adjusted incidence rate ratio (95% CI)**†
**Full period of observation (July, 2014, to April, 2015)**								
Control		2000	959	762	40	2·00	15·2	1
	Cluster median (IQR)	54·5 (7·0–98·5)	49·0 (45·0–50·0)	398·0 (51·0–776·0)	1 (0–2·5)	1·2 (0–2·5)	7·4 (0–19·6)	··
Intervention		1927	977	720	27	1·40	10·1	0·65 (0·25–1·65)
Cluster median (IQR)		24·0 (9·0–84·0)	49·0 (48·5–50·0)	172·0 (70·0–622·0)	0 (0–2)	0 (0–1·6)	0 (0–14·5)	p=0·36
**First 3 months of IPT (July to September, 2014)**								
Control		834	300	1015	18	2·16	21·9	1
	Cluster median (IQR)	11·0 (1·5–34·0)	15·0 (15·0–15·0)	268·0 (37·0–828·0)	0 (0–1)	0·46 (0–3·2)	24·4 (0–24·4)	··
Intervention		667	301	809	14	2·10	17·0	0·75 (0·28–2·04)
	Cluster median (IQR)	7·5 (3·5–27·0)	15·0 (15·0–15·0)	183·0 (88·0–657·0)	0 (0–1)	0 (0–3·1)	0 (0–24·4)	p=0·58
**Final 3 months of IPT (October to December, 2014)**								
Control		490	258	694	13	2·65	18·4	1
	Cluster median (IQR)	15·5 (1·0–30·0)	14·0 (10·0–15·0)	426·0 (37·0–790·0)	0 (0–1)	0 (0–3·4)	0 (0–39·1)	··
Intervention		467	277	616	5	1·07	6·6	0·36 (0·10–1·33)
	Cluster median (IQR)	8·0 (1·0–23·0)	14·5 (14·0–15·0)	195·0 (26·0–583·0)	0 (0–0)	0 (0–0)	0 (0–0)	p=0·13
**After IPT (January to April, 2015)**								
Control		676	401	616	9	1·33	8·2	1
	Cluster median (IQR)	21·0 (4·5–48·5)	20·0 (20·0–20·0)	384·0 (82·0–886·0)	0 (0–1·0)	0 (0–1·0)	0 (0–18·3)	··
Intervention		793	399	726	8	1·01	7·3	0·85 (0·24–3·03)
	Cluster median (IQR)	7·5 (3·5–46·5)	20·0 (20·0–20·0)	137·0 (65·0–849·0)	0 (0–0)	0 (0–0)	0 (0–0)	p=0·80

aEIR=annual entomological inoculation rate. IPT=intermittent preventive treatment.

* Calculated from the household density of infective anophelines.

† Adjusted for the main materials of the floor (cement or concrete, earth or sand, earth and dung, or other) and external walls (burnt bricks with plaster or other) and cluster.

## Discussion

School-aged children are important contributors to the human infectious malaria reservoir.^11^ Given the likelihood of operational success and sustainability of school-based interventions, the question of whether IPT of schoolchildren can reduce malaria transmission at the population level is highly relevant. In this cluster-randomised trial, monthly IPT with DP provided to children in primary schools in a population with high insecticide-treated net coverage was associated with reductions in measures of malaria transmission, including parasite prevalence in community residents and sporozoite rate, compared with in the control group, although these differences were of borderline significance. However, given that coverage (43%) was substantially lower than desired targets, they are notable. Additional studies of IPT in schoolchildren to investigate the community-level effects with greater IPT coverage are warranted.

Despite an erratic history, chemoprevention is likely to be an important addition to existing malaria control strategies, partly because of renewed interest in treating asymptomatic individuals to reduce transmission. Chemoprevention can be delivered in various ways to communities, including mass or intermittent screening and treatment, mass drug administration, and IPT of specific high-risk populations. Mass drug administration reduces malaria parasitaemia and transmission,^18^ and WHO recommends mass drug administration in specific settings, including elimination campaigns and epidemics, and to control the spread of drug-resistant parasites.^19^ A study of the short-term effects of two rounds of mass drug administration with DP in Zambia showed community-wide reductions in parasite prevalence, particularly in low-transmission areas.^20^ Nevertheless, the effects of mass drug administration are temporary unless treatment is repeated or delivered in the context of highly effective vector control,^21^ raising concerns about feasibility and sustainability. The potential for mass drug administration, and to a lesser degree other drug-based interventions, to suppress naturally acquired immunity and contribute to rebound infections and to accelerate the development of drug resistance, are other concerns.^22^ Studies of mass screening and antimalarial treatment to individuals who tested positive for malaria infection in Burkina Faso and Zambia had little effect,^23^, ^24^ due partly to the poor sensitivity of diagnostic tools available for use in field trials.^19^ By contrast, IPT of young children, which is highly effective against morbidity and mortality and has been operationalised as seasonal malaria chemoprevention in west Africa, has had a dual effect: in Senegal, chemoprevention delivered to children up to age 10 years reduced the incidence of malaria in children in the wider community by 60% and in older residents by 26%. This strategy might, therefore, have a role in transmission reduction, particularly if the age range of recipients is extended.^25^ The results from our trial contribute to the growing body of evidence of the effectiveness of IPT for malaria in older children,^7^, ^8^, ^9^, ^10^ and suggest that reducing the infectious reservoir could have positive effects on the surrounding community. Operationally, IPT of schoolchildren is likely to be more feasible than community-based delivery programmes, and should be considered as a strategy for malaria control in school-aged children and a sustainable approach to reduce transmission. Further research is needed to explore feasibility and effects on larger scales.

Achieving high coverage is key to the success of chemoprevention programmes.^18^ In this study, coverage was well below the target. Several factors contributed to the low uptake of the intervention, including community perceptions about IPT and the multistep informed consent process. We sensitised key stakeholders before recruiting study participants by meeting with individuals from health and education sectors at the national and district levels. We also established a community advisory board that included representatives from across Jinja district. Nevertheless, we found recruitment of children in the 42 intervention schools difficult. Screening was done over at least two sessions, first with parents at parent-teacher association meetings (or at home), followed by interviews with students at school. The parent-teacher association meetings were poorly attended because parents were often at work, and parents or guardians were frequently unavailable for home visits and were difficult to trace. This experience is similar to that in another school-based study done in Kenya.^26^ Additionally, some community members were initially suspicious of the IPT intervention and the motives behind it, as has been seen in other studies.^18^, ^26^ Intensive community engagement was needed to address these attitudes, which prolonged recruitment but increased success. Intervention coverage increased throughout the study, and might be improved in future research projects and IPT programmes by streamlining the consent process and including dialogue with community members to improve understanding of their perspectives and concerns and to address tensions and rumours.^27^ In a pilot study in Malawi, the addition of malaria treatment to an existing mass drug administration programme for neglected tropical diseases was well received, and coverage among children was 87%.^28^ In Uganda, and potentially in other countries, if IPT of schoolchildren were to be scaled up to a national programme with support of the Ministries of Health and Education, community perception and intervention coverage might be improved and the challenges of obtaining consent minimised.^28^

We saw no effect of IPT on the secondary outcomes, including prevalence of parasitaemia assessed by microscopy and LAMP, gametocytaemia, or anaemia. Our approach to selecting samples for LAMP assessments, which was done in only 25% of samples negative on microscopy in children younger than 5 years and 50% of negative samples in those older than 15 years, might have affected our results. Although we accounted for our sampling approach in our analysis of the data, the selected samples might not have been representative of the population overall. The lack of effect of IPT on low-density infections identified by LAMP might also reflect that IPT works by preventing incident infections rather than by treating chronic low-density infections. Gametocytaemia was determined from thick blood smears, which is less sensitive than molecular amplification of specific sexual stage genes,^29^ but we did not have the resources to do molecular tests. IPT of schoolchildren with amodiaquine and sulfadoxine-pyrimethamine in Kenya and Mali substantially reduced the prevalence of anaemia,^9^, ^30^ but in Uganda, anaemia is primarily seen in children younger than 5 years. The lack of effect on anaemia in this study is unsurprising with the low coverage of IPT and the multifactorial nature of anaemia, in which helminth infections and poor nutrition are likely to be important contributing factors.^31^

This study had several other limitations. First, because of the challenges related to recruitment, the number of children participating in the intervention group increased gradually. Ideally, enrolment would have been completed before IPT was started to enable high coverage for all six rounds of treatment. Second, attendance at cluster schools by children in the school catchment areas was surprisingly low, possibly because of travel to school and absenteeism. In 2006, 82% of primary-school-aged children in Uganda were reported to attend school, but absenteeism, particularly in rural areas, was an issue.^32^ Clear mapping of catchment areas of schools would facilitate improved assessment of intervention effects. Third, we opted not to provide a placebo to children enrolled in the control schools, which might have affected treatment-seeking behaviour of schoolchildren or community members. Fourth, our approach to microscopy (examining slides at up to 100 high-power fields before classifying them as negative) could have lacked sensitivity for low-density infections. Examinations at magnifications of up to 200 or 500 high-power fields might increase sensitivity. Finally, we opted to exclude girls at menarche in attempt to avoid administering DP in early pregnancy. Although this approach had operational advantages, it also denied these girls the potential health benefits of IPT and had the unexpected consequence of raising suspicion within the community regarding the reasons for excluding older girls.

Targeted IPT of schoolchildren with DP as an approach to malaria control at the community level in Jinja district, Uganda, might have a positive effect on malaria indicators. Coverage in our study, however, was lower than expected and differences between groups were only of borderline significance. This study makes an important contribution to the evidence for the use of IPT as a tool to reduce malaria transmission. Schoolchildren are major contributors to the infectious reservoir. In high transmission areas, innovative approaches to malaria control will be needed to reduce and ultimately eliminate the burden of malaria. IPT of schoolchildren offers an operationally attractive and potentially sustainable intervention that could be integrated with currently deployed malaria-control methods. Future studies should investigate how to achieve high coverage, methods to integrate IPT with other school-based programmes and control measures, the potential effects on naturally acquired immunity and risk of rebound, and the risk of accelerating antimalarial drug resistance.
