# Supplementary material for: Assessment of community-level effects of intermittent preventive treatment for malaria in schoolchildren in Jinja, Uganda (START-IPT trial): a cluster-randomised trial
Source: Lancet Glob Health. 2018 Apr 13;6(6):e668–79. doi: 10.1016/S2214-109X(18)30126-8 (PMC5952991; doi:10.1016/S2214-109X(18)30126-8)
Supplement: Supplementary appendix [file mmc1.pdf]

THE LANCET
Global Health

Supplementary appendix

This appendix formed part of the original submission and has been peer reviewed. We post it as supplied by the authors.

Supplement to: Staedke SG, Maiteki-Sebuguzi C, Rehman AM, et al. Assessment of community-level effects of intermittent preventive treatment for malaria in schoolchildren in Jinja, Uganda (START-IPT trial): a cluster-randomised trial. *Lancet Glob Health* 2018; published online April 13. http://dx.doi.org/10.1016/S2214-109X(18)30126-8.

Supplemental File 1

Determination of clusters and details of randomisation

Enumeration

Primary schools were mapped using hand-held GPS receivers (Garmin eTrex Legend H®), and households were enumerated manually using digital maps downloaded from Google Earth (http://www.google.com/earth/index.html).

Boundaries for Jinja district, and the location of the 218 primary schools, were exported from geographic information systems 'shapefile' format (ArcGIS 10·1, ESRI) to KMZ (keyhole mark-up language zipped) and were superimposed in Google Earth. Structures close to primary schools that were suspected to be households were identified, and the Euclidean distance between the structure and the school was calculated.

Figure. Determination of schools and clusters for inclusion in the trial

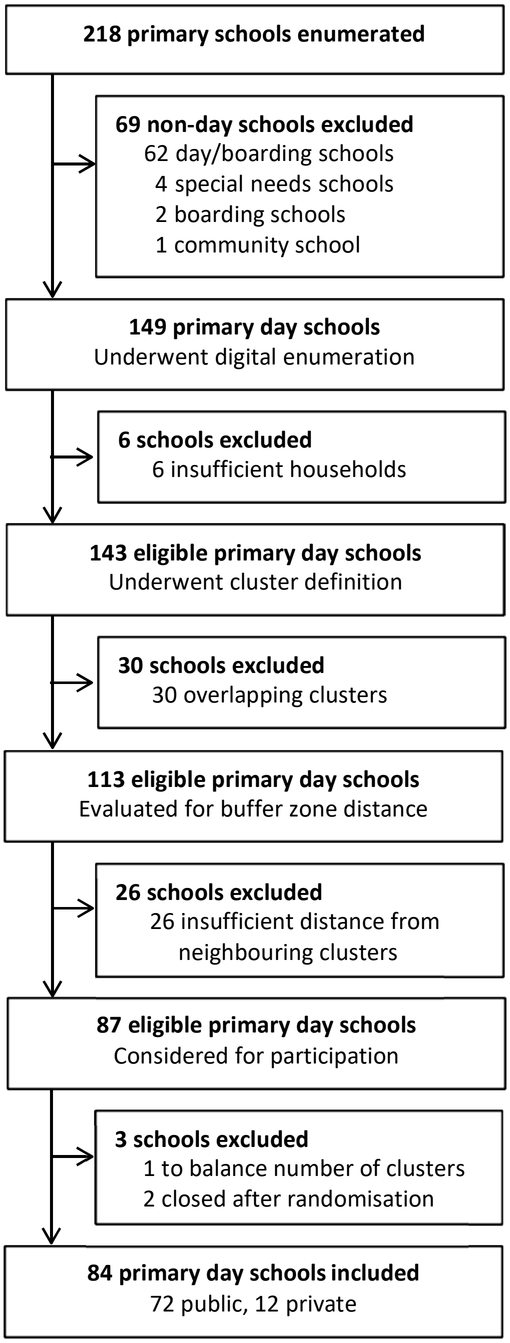

Supplemental File 2

DP Weight-based dosing guidelines – Full-strength tablets

Weight (kg)	Dihydroartemisinin-piperaquine (DP 40mg/320mg)						
	Day 0 (tablets)	Day 1 (tablets)	Day 2 (tablets)	Total DHA dose (mg/kg)	Difference = Total - Goal (6.4)	Total PQ dose (mg/kg)	Difference = Total - Goal (51.2)
11	1.00	1.00	1.00	10.9	4.5	87.3	36.1
12	1.00	1.00	1.00	10.0	3.6	80.0	28.8
13	1.00	1.00	1.00	9.2	2.8	73.8	22.6
14	1.00	1.00	1.00	8.6	2.2	68.6	17.4
15	1.00	1.00	1.00	8.0	1.6	64.0	12.8
16	1.00	1.00	1.00	7.5	1.1	60.0	8.8
17	1.00	1.00	1.00	7.1	0.7	56.5	5.3
18	1.00	1.00	1.00	6.7	0.3	53.3	2.1
19	1.00	1.00	1.00	6.3	-0.1	50.5	-0.7
20	1.00	1.00	1.00	6.0	-0.4	48.0	-3.2
21	1.50	1.50	1.50	8.6	2.2	68.6	17.4
22	1.50	1.50	1.50	8.2	1.8	65.5	14.3
23	1.50	1.50	1.50	7.8	1.4	62.6	11.4
24	1.50	1.50	1.50	7.5	1.1	60.0	8.8
25	1.50	1.50	1.50	7.2	0.8	57.6	6.4
26	1.50	1.50	1.50	6.9	0.5	55.4	4.2
27	1.50	1.50	1.50	6.7	0.3	53.3	2.1
28	1.50	1.50	1.50	6.4	0.0	51.4	0.2
29	1.50	1.50	1.50	6.2	-0.2	49.7	-1.5
30	1.50	1.50	1.50	6.0	-0.4	48.0	-3.2
31	2.00	2.00	2.00	7.7	1.3	61.9	10.7
32	2.00	2.00	2.00	7.5	1.1	60.0	8.8
33	2.00	2.00	2.00	7.3	0.9	58.2	7.0
34	2.00	2.00	2.00	7.1	0.7	56.5	5.3
35	2.00	2.00	2.00	6.9	0.5	54.9	3.7
36	2.00	2.00	2.00	6.7	0.3	53.3	2.1
37	2.00	2.00	2.00	6.5	0.1	51.9	0.7
38	2.00	2.00	2.00	6.3	-0.1	50.5	-0.7
39	2.00	2.00	2.00	6.2	-0.2	49.2	-2.0
40	2.00	2.00	2.00	6.0	-0.4	48.0	-3.2
41	3.00	3.00	3.00	8.8	2.4	70.2	19.0

Supplemental File 3

Sample size calculations: Baseline community survey

Sample size calculations for the baseline community survey were based on the primary outcome, the prevalence of parasitaemia in the community, using preliminary PRISM data from the 2013 surveys (Table 1).

Table 1. PRISM community and school survey results, Jinja district, 2012-2013

	**Community survey**			**School survey**
	**< 5 years**	**5-15 years**	**> 15 years**	**6-15 years**
**2012**				
Number	155	214	262	300
Anaemia category				
< 8.0 gm/dL	3 (1.9%)	1 (0.5%)	3 (1.2%)	0
8.0-10.9 gm/dL	60 (38.7%)	28 (13.1%)	16 (6.1%)	31 (10.3%)
11.0-11.9 gm/dL	44 (28.4%)	51 (23.8%)	25 (9.5%)	74 (24.7%)
> 12.0 gm/dL	48 (31.0%)	134 (62.6%)	218 (83.2%)	195 (65.0%)
RDT positive	30 (19.4%)	58 (27.1%)	36 (13.7%)	63 (21.0%)
Blood smear (BS) positive	13 (8.4%)	41 (19.2%)	18 (6.9%)	48 (16.0%)
Gametocytaemia	4 (2.6%)	9 (4.2%)	2 (0.8%)	7 (2.3%)
**2013**				
Number	130	150	255	300
Anaemia category				
< 8.0 gm/dL	2 (1.5%)	2 (1.3%)	1 (0.4%)	1 (0.3%)
8.0-10.9 gm/dL	58 (44.6%)	18 (12.0%)	14 (5.5%)	9 (3.0%)
11.0-11.9 gm/dL	37 (28.5%)	29 (19.3%)	26 (10.2%)	52 (17.3%)
> 12.0 gm/dL	33 (25.4%)	101 (67.3%)	214 (83.9%)	238 (79.3%)
RDT positive	35 (26.9%)	53 (35.3%)	95 (37.3%)	129 (43.0%)
Blood smear (BS) positive	5 (3.9%)	25 (16.7%)	22 (8.6%)	34 (11.3%)
Gametocytaemia	3 (2.3%)	6 (4.0%)	4 (1.6%)	5 (1.7%)

The prevalence of parasitaemia was assumed to vary with age, with baseline estimates of 4% in children under five, 17% in children aged 5-15 years, and 9% in adults > 15 years. We aimed to detect a relative reduction of 35% in the intervention arm as compared to the control, in each age group, and 22% overall, as shown in Table 2.

Table 2. Number of participants required per cluster, for baseline community surveys

	**BASELINE Survey**			
**Age strata**	**Parasite prevalence Control**	**Relative reduction**	**Parasite prevalence DP arm**	**Participants per cluster (84 clusters)**
**< 5 years**	4%	0.35	2.6%	73
**5-15 years**	17%	0.35	11.1%	15
**> 15 years**	9%	0.35	5.9%	31
**Total**	10%	0.22	7.8%	119

We aimed to test the primary hypothesis that community residents living in clusters surrounding schools randomised to the intervention would have a lower prevalence of parasitaemia than those living in near schools randomised to control. We planned to include 84 clusters in the study, and to sample 119 residents per cluster (9,996 total).

With two study arms, half of the clusters assigned to each study arm, and a coefficient of variation between clusters of 0.25, we estimated that surveying 119 residents in each cluster would allow us to detect a relative difference in parasite prevalence of 22% (or more) between the study arms in the total population, with power of 80% and significance of 5%.[1] This relative difference in parasitaemia corresponds to an absolute difference in parasite prevalence of 2.2% (10% versus 7.8%). In addition, for each of the age groups, the sample size was weighted to give 80% power to detect a relative difference in parasite prevalence of 35% between the study arms (Table 2).

Sample size calculations: Baseline school survey

For the primary outcome of parasitaemia measured in baseline school surveys, we assumed a prevalence of 11% in the control group based on recent data from PRISM school surveys conducted in 2013 in Jinja (Table 1). We planned to survey 96 randomly selected children in each of the 84 clusters (8,064 total), which would give over 80% power at significance level 5% to detect a relative reduction in parasite prevalence of 22%, corresponding to an absolute difference in parasite prevalence of 2.4% (11% versus 8.58%), assuming a coefficient of variation between clusters of 0.25 (Table 3).

Table 3. Number of participants required per cluster, for baseline school surveys

	**BASELINE Survey**			
**Age strata**	**Parasite prevalence Control**	**Relative reduction**	**Parasite prevalence DP arm**	**Participants per cluster (84 clusters)**
**Total**	11%	0.22	8.58%	96

References

1. Hayes R, Bennett S. Simple sample size calculations for cluster-randomized trials. *International Journal of Epidemiology* 1999; **28**(2): 319-26.
